# Editorial: Platelet Rich Plasma (PRP) in Companion and Farm Animals

**DOI:** 10.3389/fvets.2021.834546

**Published:** 2022-02-07

**Authors:** Roberta Perego, Daniela Proverbio, Jorge U. Carmona

**Affiliations:** ^1^Department of Veterinary Medicine, Faculty of Veterinary Medicine, University of Milan, Milan, Italy; ^2^Grupo de Investigación Terapia Regenerativa, Departamento de Salud Animal, Universidad de Caldas, Manizales, Colombia

**Keywords:** platelet-related hemocomponents, dog, cat, horse, skin wounds, corneal ulcers, bacteria control, platelet-rich plasma (PRP)

Platelet-rich plasma (PRP) an hemocomponent for non-transfusional use has gained an important popularity and research interest during last years, as a treatment for multiple conditions in both human and veterinary medicine (1). This simple, versatile, and very cheap biomaterial was initially employed for improving outcomes in human patients with alveolar maxillary defects (2, 3) and then its clinical usage irradiated many specialties of the human and veterinary medicine.

Currently, it is well recognized that PRP, as well as another related hemocomponents, have regenerative, anti-inflammatory and bacteriostatic properties, which begin to be rutinary used for veterinary practitioners around the world in diverse fields, such as dermatology, ophthalmology and sports medicine, among others (4). However, several questions remain unsolved regarding what is the ideal concentration of platelets and leukocytes in PRP, what type of PRP is the most indicated for the treatment of a particular condition, what is the better scheme of administration, and so forth (5). For sure, these questions will be solved with the research progress in this amazing research field.

In line with this, we present a collection on PRP in companion animals and farm animals of four articles, three of which three are original researches on dogs and cats and one is a literature review on horses. The effect of PRP on canine skin wounds was investigated by Iacopetti et al. in “Autologous Platelet-Rich Plasma Enhances the Healing of Large Cutaneous Wounds in Dogs.” This clinical study evaluated the effects of two consecutive applications of autologous PRP, with the second application after 15 days, in 6 dogs showing large subacute skin wounds. The authors observed that dogs treated with this biomaterial presented a good clinical response in terms of contraction, re-epithelialization and healing with no complications related to PRP treatments, concluding this substance could represent a simple, cost-effective, and valid alternative to promote healing processes in subacute large wounds cases in dogs.

In the study “Corneal Ulcer in Dogs and Cats: Novel Clinical Application of Regenerative Therapy Using Subconjunctival Injection of Autologous Platelet-Rich Plasma,” Farghali et al. evaluated the capacity of the subconjunctival injection of autologous PRP in the treatment of corneal ulcers in small animals and determined the expression of matrix metalloproteinase (MMP)-2, MMP-9, and oxidative stress biomarkers in tears of the treated patients. 28 animals (16 cats and 12 dogs) with corneal ulcers of different etiology, including feline herpes virus type 1 infections in cats, were enrolled in the study. They found that 50% of dogs needed two injections with 1-week intervals and 50% of cats needed three injections with 1-week intervals for corneal ulcers healing. Furthermore, tear oxidative markers and matrix metalloproteinases were reduced by the PRP effect. The authors concluded the subconjunctival injection PRP is a relatively cheap, safe, and effective treatment for corneal ulcers in dogs and cats.

The *in vitro* bactericidal effects of several canine platelet-related hemocomponents were evaluated by Attili et al. in “Antibacterial Properties of Canine Platelet-Rich Plasma and Other Non-Transfusional Hemo-Components: An in vitro Study.” In this study the authors evaluated the potential antibacterial properties of several canine platelet-related formulations (platelet-rich plasma, platelet gel, platelet lysate, fibrin glue), considering both leukocyte-rich and leukocyte-poor products, but also platelet-poor plasma and activating substances (thrombin, calcium gluconate) against *Staphylococcus aureus subspecies aureus, Staphylococcus cohnii subspecies cohnii, Escherichia coli, Pseudomonas aeruginosa, and Klebsiella pneumoniae subspecies pneumoniae*. They evaluated the effect of these substances by using agar gel diffusion method (Kirby–Bauer) and micro-inhibition in broth using microplates and spectrophotometer reading and found these hemocomponents have a bacteriostatic effect against Gram-negative bacteria, independent of the concentration of platelets and leukocytes in the evaluated hemocomponents. This study concluded that non-transfusional hemocomponents could be a useful in controlling bacterial infections in dogs.

A review about the PRP biology and its clinical use in horses was presented by Camargo-Garbin et al. in “A Critical Overview of the Use of Platelet-Rich Plasma in Equine Medicine Over the Last Decade.” In this work the authors made a narrative review of the most significant aspects related with the basic mechanisms affecting tissue response to PRP in horses and how a plethora of platelet-related products are being used in equine medicine and surgery. The authors concluded that although PRP is increasingly used in equine practice, even persist lack of qualitative and quantitative evidence-based data supporting PRP's clinical use in this species, despite of the numerous studies intended to accomplish this purpose.

## Author Contributions

All authors listed have made a substantial, direct, and intellectual contribution to the work and approved it for publication.

## Conflict of Interest

The authors declare that the research was conducted in the absence of any commercial or financial relationships that could be construed as a potential conflict of interest.

## Publisher's Note

All claims expressed in this article are solely those of the authors and do not necessarily represent those of their affiliated organizations, or those of the publisher, the editors and the reviewers. Any product that may be evaluated in this article, or claim that may be made by its manufacturer, is not guaranteed or endorsed by the publisher.
